# Therapeutic Effect of Exogenous Truncated IK Protein in Inflammatory Arthritis

**DOI:** 10.3390/ijms18091976

**Published:** 2017-09-14

**Authors:** Seulgi Choi, HyeLim Park, SeoYeon Jung, Eun-Kyung Kim, Mi-La Cho, Jun-Ki Min, Su-Jin Moon, Sang-Myeong Lee, Jang-Hee Cho, Dong-Hee Lee, Jae-Hwan Nam

**Affiliations:** 1 Department of Biotechnology, The Catholic University of Korea, Bucheon 14662, Korea; cjscld3@naver.com (S.C.); csphl@naver.com (H.P.); jssry@naver.com (S.J.); 2 The Rheumatism Research Center, Catholic Research Institute of Medical Science, College of Medicine, The Catholic University of Korea, Seoul 06591, Korea; mnikek@catholic.ac.kr (E.-K.K.); iammila@catholic.ac.kr (M.-L.C.); 3 Division of Rheumatology, Department of Internal Medicine, College of Medicine, The Catholic University of Korea, Seoul 06591, Korea; min6403@catholic.ac.kr (J.-K.M.); prajna79@catholic.ac.kr (S.-J.M.); 4 Department of Biotechnology, Chonbuk National University, Iksan 54596, Korea; leesangm@jbnu.ac.kr; 5 Biomaterials Research Center, Cellinbio, Suwon 16680, Korea; cjh@cellinbio.co.kr (J.-H.C.); leedh@cellinbio.co.kr (D.-H.L.)

**Keywords:** autoimmune disease, rheumatoid arthritis, T cells, truncated IK

## Abstract

Inhibitor K562 (IK) protein was first isolated from the culture medium of K562, a leukemia cell line. It is known to be an inhibitory regulator of interferon-γ-induced major histocompatibility complex class (MHC) II expression. Previously, we found that transgenic (Tg) mice constitutively expressing truncated IK (tIK) showed reduced numbers of pathogenic Th1 and Th17 cells, which are known to be involved in the development of rheumatoid arthritis (RA). Here, we investigated whether exogenous tIK protein has a therapeutic effect in arthritis in disease models and analyzed its mechanism. Exogenous tIK protein was produced in an insect expression system and applied to the collagen antibody-induced arthritis (CAIA) mouse disease model. Injection of tIK protein alleviated the symptoms of arthritis in the CAIA model and reduced Th1 and Th17 cell populations. In addition, treatment of cultured T cells with tIK protein induced expression of A20, a negative regulator of nuclear factor-κB (NFκB)-induced inflammation, and reduced expression of several transcription factors related to T cell activation. We conclude that exogenous tIK protein has the potential to act as a new therapeutic agent for RA patients, because it has a different mode of action to biopharmaceutical agents, such as tumor necrosis factor antagonists, that are currently used to treat RA.

## 1. Introduction

Inhibitor K562 (IK) was first isolated from the culture medium of K562, a leukemia cell line. It is known to be a downregulator of interferon-γ (IFN-γ)-induced major histocompatibility complex (MHC) class II, which functions as an important factor in the immune response [1,2,3]. Previous studies suggest that the function of IK may be associated with the class II transactivator (CIITA), which is an influential player in both constitutive and cytokine-inducible expressions of MHC class II, because excess expression of IK negatively regulated CIITA mRNA transcription and reduced the level of expressed MHC class II [4]. In addition, we reported that Coxsackievirus B3 (CVB3) infection transiently induced IK mRNA and that the expressed IK protein bound to G protein-coupled receptors and increased cyclic AMP (cAMP) levels in cytoplasm, and that this CVB3-IK-cAMP axis was the cellular mechanism behind the previous observation that CVB3 infection reduced MHC class II expression in myocytes [5]. Therefore, induction of IK might be a survival strategy of CVB3 to escape host immune responses [6].

Abnormal expression and uncontrolled function of MHC class II have been recognized as one factor in the development of autoimmune diseases [7,8,9]. Because IK can regulate MHC class II expression, it may have therapeutic potential in autoimmune diseases. One study used truncated IK (tIK) missing a 315-amino acid sequence from the N-terminus in a study of lupus nephritis [10], and showed that tIK and full-length IK reduced IFN-γ-induced MHC class II expression to a similar extent. Moreover, tIK-treated mice showed reduced kidney damage and decreased infiltration of immune cell populations into their kidneys [10].

Our previous study demonstrated the effects of tIK on rheumatoid arthritis (RA) using three mouse models: interleukin (IL)-1 receptor antagonist knockout (IL1RaKO) mice, an autologous induced RA model, and IL1RaKO/tIK-transgenic mice, termed tIK-IL1RaKO mice [11]. We observed milder symptoms and lower severity of RA in tIK-IL1RaKO mice compared with IL1RaKO mice. Moreover, tIK had a downregulatory effect on CD4^+^ T helper cells producing IFN-γ and IL-17 (Th1 and Th17 cells), which are responsible for the pathology of RA. When splenocytes isolated from wild-type (WT) Balb/c mice and tIK-transgenic (tIK-Tg) mice were polarized in vitro into Th17 cells, the number of Th17 cells produced from tIK-Tg splenocytes was lower than that from WT Balb/c splenocytes. Moreover, macrophages isolated from tIK-Tg mice were resistant to lipopolysaccharide (LPS)-induced inflammation [11].

Th17 cells and the IL-17 signaling pathway are important in the induction of autoimmune disease. Because of this, they have been highlighted as therapeutic candidates [12,13,14,15]. Although IK can inhibit the differentiation of Th17 cells, it is not clear how IK downregulates this process and thereby reduces the severity of arthritis in disease models. Moreover, our previous study used tIK-Tg mice, which endogenously express tIK, to demonstrate the therapeutic effect of tIK in inflammatory arthritis [11]. Therefore, in this study, we investigated the possibility of using exogenous tIK protein as a therapeutic agent for inflammatory arthritis, and the factors associated with its therapeutic effect. Using the collagen antibody-induced arthritis (CAIA) mouse model, although it has some limitations, we demonstrated that mice injected with exogenous tIK protein showed alleviation of arthritis severity and a reduction in T cell differentiation, similar to that observed in tIK-Tg mice endogenously expressing tIK. Moreover, treatment with tIK protein induced expression of A20, a negative regulator of the NFκB pathway, in splenic CD4^+^ T cells from WT mice. These findings suggest that exogenous tIK protein may have potential as an effective therapeutic agent for inflammatory autoimmune diseases including RA.

## 2. Results

### 2.1. Exogenous tIK Protein Is Expressed in an Insect Cell Culture System

**Figure 1:**
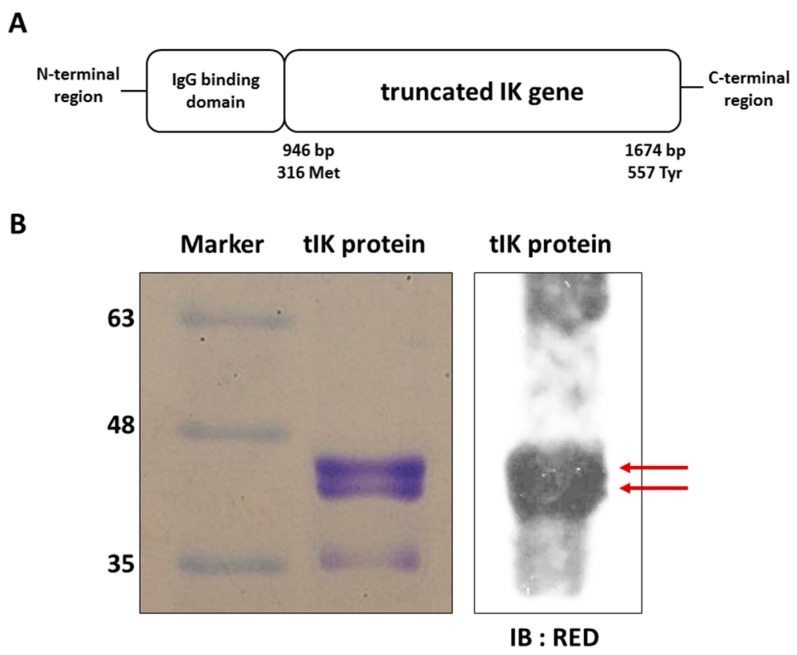
Exogenous truncated IK (tIK) protein is expressed in an insect cell culture system. (**A**) Diagram of the immunoglobulin G (IgG) binding domain-tagged tIK protein with nucleotide and amino acid sequence numbers was generated according to reference [6]; (**B**) Purified tIK protein was stained by Coomassie blue after SDS-PAGE (left). The size of the tIK protein was predicted to be approximately 40 kDa. tIK protein was also detected by Western blotting (right) using RED-specific primary antibody, which recognizes a unique amino acid sequence located in tIK. The red arrows indicate the expected tIK protein.

We produced the exogenous tIK protein from cultured *Spodoptera frugiperda* Sf9 insect cells. The tIK comprised residues from methionine 315 to tyrosine 557 of the full-length IK protein fused with the immunoglobulin G (IgG) binding domain for purification and secretion (Figure 1A). Its nucleotide and amino acid sequences are shown in Figure S1. The size and purity of the tIK protein was assessed by SDS-PAGE (Figure 1B), which showed two bands around 40–45 kDa, the expected size of the expressed tIK protein. The identity of the protein was confirmed by Western blotting using a specific antibody for the RED region, a unique amino acid sequence within IK protein [16] (Figure 1B).

### 2.2. Treatment with tIK Protein Prevents the Differentiation of Naïve CD4^+^ T Cells into Th17 Cells

**Figure 2:**
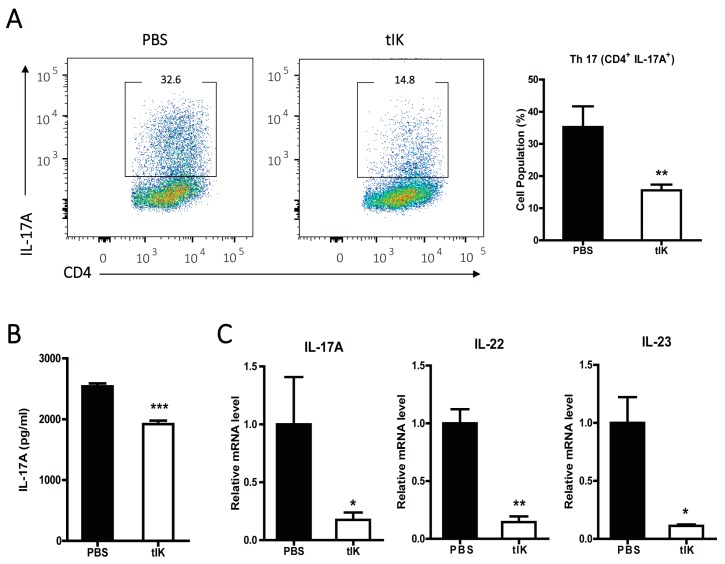
Treatment of naïve CD4^+^ T cells with tIK protein suppresses their differentiation into Th17 cells. (**A**) CD4^+^ T cells isolated from 7-week-old Balb/c mice using magnetic activated cell sorting were cultured in Th17-polarizing medium. After 3 days, the differentiated cells were collected and stained with anti-CD4-APC and anti-IL-17A-PE for analysis using flow cytometry. The pseudo-color dot plots represent cell distribution in CD4^+^ T cells and the cells gated in the box express IL-17. Therefore, the cells in the box are Th17 cells (CD4^+^ IL-17A^+^). The mean percentages of Th17 cells are shown by the black bar graph; (**B**) Stimuli (anti-CD3 and anti-CD28 antibodies (each 1 μg/mL)) were added to the Th17-differentiated cells. After 24 h incubation, the culture supernatant was harvested and the concentration of IL-17A in the supernatant was quantitated using ELISA; (**C**) The mRNA levels for IL-17, IL-22 and IL-23 in the Th17-differentiated cells shown in Figure 2A were analyzed by qPCR. All experiments were independently repeated three times. Data is represented as the mean ± SD (*n* = 3), * *p* < 0.05, ** *p* < 0.005, *** *p* < 0.001.

To investigate the effect of tIK protein on Th17 cell differentiation, we treated naive CD4^+^ T cells isolated from the spleens of WT mice with exogenous tIK protein under Th17-polarizing conditions. We observed cell clumps in the Th17-polarizing conditions but not in the normal culture medium (Figure S2). We analyzed the resulting cells using flow cytometry after intracellular staining to measure the Th17 cell population. Compared with cells treated with phosphate-buffered saline (PBS) in Th17-polarizing medium, the tIK protein-treated cells showed reduced Th17 cell differentiation (Figure 2A). Moreover, the level of IL-17A secreted from Th17 cells after they were stimulated with anti-CD3 and anti-CD28 antibodies for 24 h was lower in tIK protein-treated cells than in controls (Figure 2B). The levels of mRNA for IL-17A and other cytokines associated with Th17 cells including IL-17A, IL-22, and IL-23, as measured by qPCR, were also lower in tIK protein-treated cells than in PBS-treated cells (Figure 2C).

### 2.3. Treatment with tIK Protein Suppresses the Production of Proinflammatory Cytokines after CD4^+^ T Cell Activation

**Figure 3:**
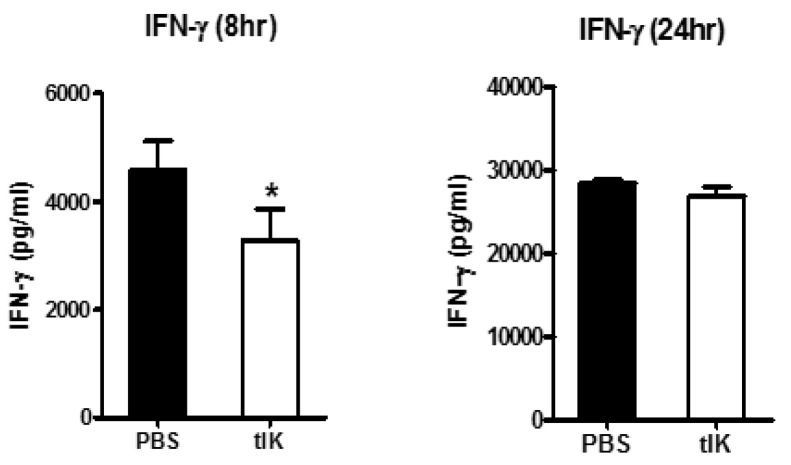
Treatment with tIK protein suppresses the production of pro-inflammatory cytokine, IFN-γ, during CD4^+^ T cell activation. CD4^+^ T cells isolated from 7-week-old Balb/c mice using magnetic activated cell sorting were activated with anti-CD3 and anti-CD28 antibodies (each 1 μg/mL) for 8 h and 24 h. Phosphate-buffered saline (PBS) or tIK protein was added to the cells at the same time as treatment with anti-CD3 and anti-CD28 antibodies. The concentrations of IFN-γ in the supernatants were measured by ELISA. Data is represented as the mean ± SD (*n* = 3), * *p* < 0.05.

We also investigated the effect of tIK protein on activation of CD4^+^ T cells. Typically, activated CD4^+^ T cells can produce IFN-γ [17]. Therefore, we measured the expression level of this cytokine secreted by CD4^+^ T cells at 8 h and 24 h after stimulation with anti-CD3 and anti-CD28 antibodies. As expected, tIK protein-treated T cells (tIK) clearly showed reduced production of these inflammatory cytokines compared with PBS-treated T cells (PBS) (Figure 3).

### 2.4. Treatment with tIK Protein Induces Expression of A20, a Negative Regulator of Inflammation in CD4^+^ T Cells

**Figure 4:**
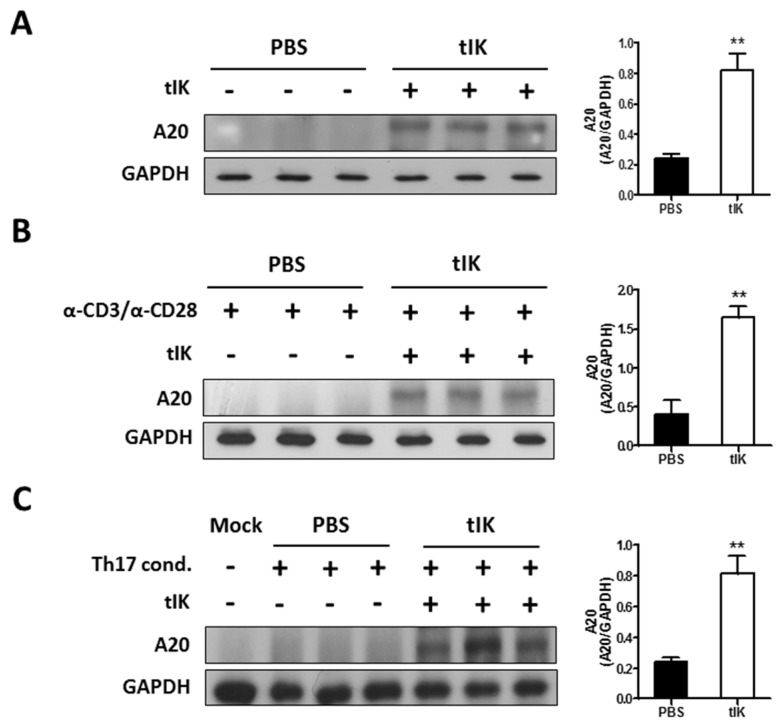
Treatment with tIK protein induces expression of A20, a negative regulator of inflammation in CD4^+^ T cells. (**A**) CD4^+^ T cells isolated from 7-week-old Balb/c mice using magnetic activated cell sorting were harvested 8 h after the tIK protein treatment and lysed using RIPA buffer containing a protease inhibitor cocktail. A20 was detected in the total extracted protein by Western blotting; (**B**) CD4^+^ T cells were isolated and stimulated with anti-CD3 and anti-CD28 antibodies (each 1 μg/mL). The cells were harvested 8 h after the stimulation and tIK protein treatment. Protein from the cells was extracted and used for Western blot analysis as described in Figure 4A; (**C**) CD4^+^ T cells were isolated and differentiated into Th17 cells in Th17-polarizing medium. Protein was extracted from the cells cultured for 3 days and used for Western blot analysis as described for Figure 4A. PBS indicates non-treated (control) cells, tIK indicates tIK protein-treated cells. The amount of protein was normalized to that of glyceraldehyde 3-phosphate dehydrogenase (GAPDH) protein. The bar graph shows mean normalized data. Data is represented as the mean ± SD (*n* = 3), ** *p* < 0.005.

A previous report showed that CD4^+^ splenic T cells from tIK-Tg mice expressed A20, a negative regulator of NFκB, under Th17-polarizing conditions [11]. In this study, we investigated the effect of exogenous tIK protein on the induction of A20 in stimulated and unstimulated T cells. The results shown in Figure 4A indicate that tIK protein treatment of T cells isolated from spleens of WT mice induced a time-dependent expression of A20; the highest expression of A20 was at 8 h post-treatment and A20 expression continued until 48 h post-treatment (Figure S3A). Moreover, tIK protein could induce A20 expression under both T cell-activating and Th17-polarizing conditions (Figure 4B,C) and A20 expression was also induced in isolated naive CD4^+^ T cells under T cell activating conditions (Figure S3B).

### 2.5. Treatment with tIK Protein Affects the Activation of Various Transcription Factors

**Figure 5:**
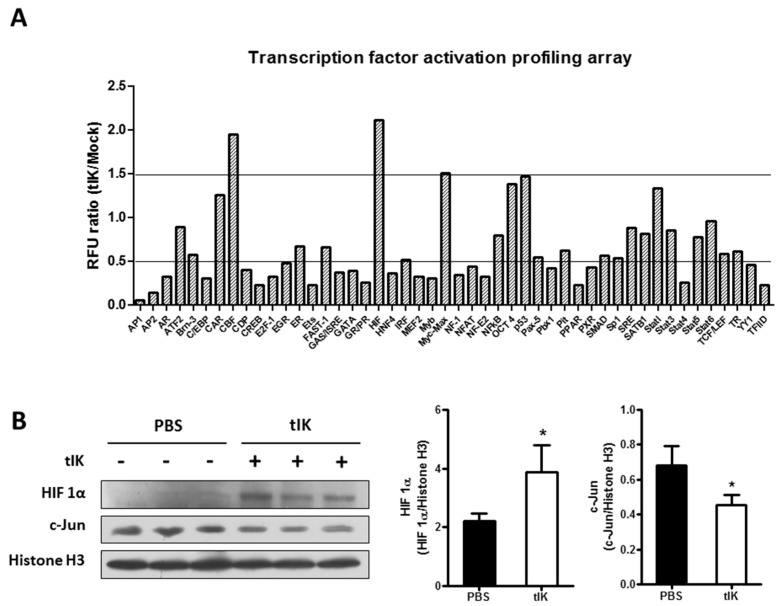
Treatment with tIK protein affects the activation of various transcription factors. (**A**) Nuclear extracts from CD4^+^ T cell treated with PBS or tIK protein for 8 h were used as samples for a plate array and the activation level of several transcription factors was compared between the PBS-treated group and the tIK protein-treated group. The RFU indicates the ratio of values for transcription factors in tIK-treated and control (PBS-treated) cells; (**B**) The expression level of HIF-1α and c-Jun (AP-1 component) were confirmed by Western blotting. Nuclear extract was prepared as described for Figure 5A and used for confirmation. Data is represented as the mean ± SD (*n* = 3), * *p* < 0.05.

To examine the impact of tIK protein treatment on signal transduction pathways, we performed a plate-array analysis to monitor the activation of various transcription factors after tIK protein treatment of naïve CD4^+^ T cells isolated from WT mouse spleens. Expression of 23 transcription factors, including activator protein (AP)-1, AP-2, interferon regulatory factor (IRF), and nuclear factor of activated T cells (NFAT) was significantly reduced (by at least 50%) in tIK protein-treated cells (Figure 5), whereas that of three factors, myc-associated factor X (Myc-Max, 1.51 times), core binding factor (CBF, 1.95 times) and hypoxia inducing factor (HIF, 2.11 times), was meaningfully increased (Figure 5). However, we used a single plate array to monitor the activation of transcription factors, so were unable to calculate the statistical significance of differences. The expression of only two factors, HIF and c-Jun (one component of AP-1), was confirmed by Western blot analysis (Figure 5B). However, further analysis is required to identify the detailed mechanisms and the role of these factors in tIK protein function.

### 2.6. Exogenous tIK Protein Alleviates the Inflammatory Symptoms in a Mouse Model of Arthritis

**Figure 6:**
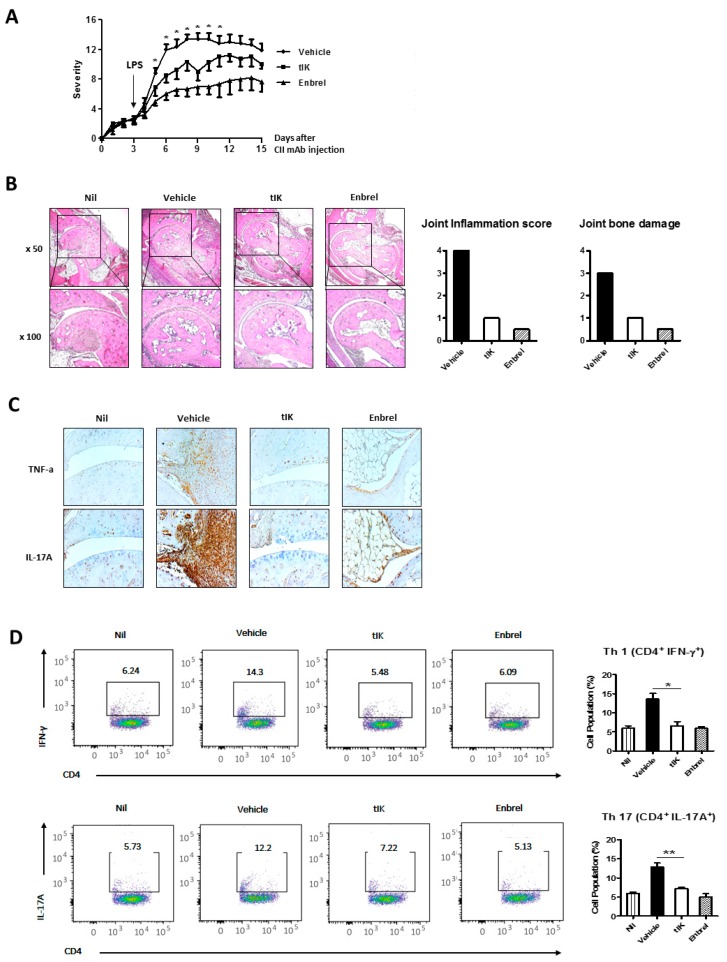
The injection of the exogenous tIK protein in a mouse model of RA alleviates the inflammatory symptoms. (**A**) The severity of arthritis in the CAIA model was scored every day. The severity of symptoms in each paw was scored on a scale from 0 (no symptoms) to 4 (maximal level of symptoms) and the scores for four limbs were summed; * *p* < 0.05, Enbrel or tIK vs. vehicle; The black arrow indicated the day of LPS treatment. (**B**) Hematoxylin and eosin (H&E) staining of the ankle joint tissue was performed to analyze tissue damage. A representative image was selected for each group (*n* = 6–9); The black square indicated the enlarged area with x100. (**C**) Immunohistochemistry staining for the inflammatory cytokines TNF-α and IL-17A was performed. A representative image for each group was selected (*n* = 6–9); (**D**) The percentages of Th1 and Th17 cells were analyzed using flow cytometry. Splenocytes were isolated and stained with anti-CD4-allophycocyanin (APC) as a surface marker and anti-IFN-γ-phycoerythrin (PE) or anti-IL-17A-PE as an intracellular marker. The pseudo-color dot plots represent cell distribution in CD4^+^ T cells and the cells gated in the box express IFN-γ or IL-17A; the bar graph shows mean percentages. Data is represented as the mean ± SD (*n* = 3), * *p* < 0.05, ** *p* < 0.005.

Based on the in vitro results shown above, we examined the effect of exogenous tIK protein in an animal arthritis model (CAIA). The injected amount of tIK protein (10 mg/kg) per mouse was chosen to be equal to the amount of Enbrel used as a positive control, because we wanted to compare the therapeutic effect of injecting the same amount of each protein, and previous experiments had shown that 10 mg/kg of Enbrel was protective in the CAIA mouse model (data not shown). CAIA in mice is a simple and powerful animal model of RA and, compared with the collagen-induced arthritis (CIA) model, rapidly induces arthritis symptoms. On average, the symptoms of arthritis appear in the CAIA model at day 3 or 4 post-injection with anti-collagen antibody and are most severe at day 8–10 post-injection, whereas in the CIA model, the symptoms of arthritis appear only 3 weeks after immunization with collagen and last for 6–8 weeks after immunization. Because not all autoantibodies generated against collagen in the CIA model induce arthritis, CAIA redresses this defect by using a cocktail consisting of several monoclonal antibodies [18,19,20]. After stimulation with LPS 0111:B4 (Chondrex, 5 μg), CAIA mice injected intraperitoneally (i.p) with 10 mg/kg of tIK protein daily showed less severe (average severity score 9.86 at day 9 post-injection) arthritis than control CAIA mice (Vehicle) injected with PBS (average severity score 13.17 at day 9 post-injection), but slightly more severe arthritis than mice treated three times at three-day intervals with Enbrel, a biopharmaceutical currently used to treat RA (average severity score 7.92 at day 9 post-injection (Figure 6A) (*p* < 0.05). The detailed schedule for these animal experiments is shown in Figure S4.

The damage to tarsal bones and the infiltration of immune cells to the joint tissue were also analyzed. Compared with control mice, tIK-injected mice showed milder bone damage and immune cell infiltration similar to that seen in Enbrel-injected mice at 10 days after CAIA induction (Figure 6B). The scores for inflammation and bone damage in tIK- and Enbrel-injected mouse joints, calculated using a previously reported scoring protocol [21], were lower than those in the vehicle group (Figure 6B). Even though technical problems meant that not all slides covered the same area of the mouse joint, the levels of inflammatory cytokines (TNF-α and IL-17A) expressed in the joint tissue were lower in tIK-injected mice and Enbrel-injected mice than in control mice (Figure 6C). However, although the overall expression pattern of proinflammatory cytokines and bone damage in the mouse joints was clearly identified, the statistical significance of differences could not be calculated because of the limitation in slide numbers. Moreover, at 10 days after induction, the numbers of IFN-γ-producing CD4^+^ T cells (Th1) and IL-17-producing CD4^+^ T cells (Th17) in tIK-treated mice were clearly reduced compared with control mice, and were similar to those in Enbrel-injected mice (Figure 6D). This flow cytometry data was obtained in the following order: after FSC/SSC and singlet gating, the cell population was finally gated by CD4^+^/IFN-γ or CD4^+^/IL-17A^+^. They were not adjusted by cell viability. Thus, the effect of dead cells was not ruled out. However, these results of the reduction of Th1 and Th17 by tIK protein were clearly in accordance with previous results [11]. Therefore, these observations suggest that exogenous tIK protein may have a suppressive effect on the induction of Th1 and Th17 cells and the inflammatory symptoms of arthritis.

## 3. Discussion

A previous report showed that tIK-Tg mice, which endogenously express tIK, showed resistance to inflammatory arthritis by suppressing differentiation of Th1 and Th17 cells and macrophage activation [11]. Thus, the purpose of this study was to determine whether treatment of WT mice with exogenous tIK protein has a similar therapeutic effect on inflammatory arthritis, and to confirm the detailed mechanism of its action. In this study, we found that treatment with exogenous tIK protein induced the expression of A20 in CD4^+^ T cells under diverse conditions including T cell activation and Th17 differentiation. A20, or tumor necrosis factor alpha-induced protein 3, restricts NFκB activation by a negative feedback through its deubiquitinase function that targets various signaling proteins dependent on NFκB stimulation [22,23,24,25]. Therefore, A20 is considered an attractive therapeutic target in inflammatory and autoimmune diseases. Delivery of A20 using a viral vector attenuated the inflammatory symptoms of RA and allergic asthma [26,27]. Thus, induction of A20 by exogenous tIK protein may be one mechanism by which the tIK induces resistance to inflammatory arthritis. In addition, exogenous tIK protein-treated CAIA mice showed reduced severity of arthritis compared with PBS-treated CAIA mice, and the Th1 and Th17 cell populations that induce inflammatory responses were lower in the spleens of tIK-treated mice compared with PBS-treated mice. These results indicate that the exogenous tIK protein produced using an insect cell culture expression system has the potential to be used as an arthritis therapy. Considering the disease model, it has been reported that the CAIA model is less dependent on B and T cells than the CIA model [28], although some reports have indicated that T cells are involved in the development of inflammatory arthritis in the CAIA model [29]. Therefore, our disease model (CAIA) has limitations in testing the effects of tIK protein, which have been previously reported as the suppression of Th17 cell differentiation and macrophage activation [11]. However, because the CAIA model has other advantages, such as a short experiment duration, it is a well-established model for arthritis and somewhat T cell dependent [29], the results obtained for tIK treatment in the CAIA model may reflect the clinical effect of tIK protein in inflammatory arthritis. In addition, in this model this weak dependence of the disease on T and B cells may be one reason why the protective effect of the tIK protein injection was not as great as that of the Enbrel injection (Figure 6A).

The therapeutic function of exogenous tIK protein for inflammatory arthritis could be explained by the reduced activation of various transcription factors associated with RA, including AP-1, IRF-4, and NFAT, that was seen in our plate-array transcription factor activation profiling analysis. AP-1 is required for T cell activation mediated by the T cell receptor and costimulatory signals [30]. The binding of AP-1 to DNA induces expression of inflammatory genes [31,32], and AP-1 is markedly increased in both RA patients and the CIA mouse model [33]. In addition, the IRF family are critical transcription factors in inflammatory processes and one of the gene families that have been implicated in susceptibility to autoimmune diseases, including RA [34,35]. In particular, IRF-4 controls the production of IL-17 and IL-21, which are expressed by Th17 cells and are involved in the pathogenesis of inflammatory arthritis [36]. Moreover, NFAT is required not only for activation but also for differentiation of T cells [37,38]. Furthermore, A20 is required for the survival and responses of T cells in vivo [39]. Thus, although in this study no data for A20 was obtained from in vivo mouse experiments, it is possible that exogenous tIK protein-induced A20 participates in controlling T cell differentiation and activation. However, further detailed in vivo studies are required to confirm the relationship between tIK protein and A20. Taken together, the reduction in the activity of these transcription factors in tIK protein-treated T cells suggests that tIK protein has anti-inflammatory and inhibitory effects during T cell activation and Th17 differentiation.

An in silico 3D structure analysis of tIK protein showed that it was similar to that of IL-10 dimer (Figure S5). Notably, the level of IL-10 is increased in bone-marrow-derived macrophages isolated from tIK-Tg mice after LPS treatment compared with those from WT mice [11]. IL-10 is a typical anti-inflammatory cytokine that acts by inhibiting the activity of various immune cells including Th1 cells, macrophages, and NK cells, but also by suppressing the expression of inflammatory genes [40,41]. In addition, IL-10 downregulates the expression of MHC class II on monocytes and reduces antigen-specific T cell proliferation [42]. Therefore, IL-10 is a key regulator of the homeostasis of the immune system and has been investigated as a candidate modulator of various chronic inflammatory diseases [43]. Based on our data, we predict that IK has a close association with IL-10 and may be part of an IL-10 subfamily. However, more detailed investigations are required to clarify the relationship between IK and IL-10.

Overall, this study has some limitations, such as the lack of statistical significance of some data and questions concerning the suitability of the CAIA model, as well as the lack of in vivo confirmation of A20. However, the results of our study showed that exogenous tIK protein has a therapeutic function in inflammatory arthritis through prevention of the activation and differentiation of CD4^+^ T cells and the induction of A20. Although exogenous tIK treatment reduced inflammatory arthritis symptoms in the CAIA model, which is an acute RA model that is driven by the complement and the innate immune systems [44,45], Enbrel, which is a recombinant TNF-α antagonist currently used as a biopharmaceutical in RA patients [46], showed greater effectiveness than tIK protein. However, Enbrel acts on downstream factors in the RA signal transduction pathway [47], while tIK protein acts on upstream factors such as T cell activation and Th17 differentiation. This means that tIK has a different mode of action to that of Enbrel, and therefore, exogenous tIK protein has the potential to act as a new therapeutic agent for patients who are resistant to Enbrel.

## 4. Materials and Methods

### 4.1. Expression and Purification of tIK Protein

To prepare tIK protein, an insect cell culture system was used. Protein expression and purification were performed by ATGene using the Invitrogen Baculo Expression Vector System (BEVS), the insect cell line Sf9, and the transfection reagent Cellfectin II (Invitrogen, Grand Island, NY, USA). The purity of the recombinant tIK protein was confirmed by Coomassie staining and Western blotting using a primary antibody specific for IK; RED (Thermo Fisher Scientific, Waltham, MA, USA).

### 4.2. Th17 Cell Differentiation

Naïve CD4^+^ T cells were isolated from the spleen of WT Balb/c mice using magnetic activated cell sorting (MACS) CD4^+^ T Cell Isolation kit (Miltenyi Biotec, Cologne, Germany) according to the manufacturer’s instructions. The RPMI medium for stimulating Th17 cell differentiation included 1 μg/mL anti-CD3 (eBioscience, San Diego, CA, USA), 1 μg/mL anti-CD28 (eBioscience), 2 ng/mL transforming growth factor (TGF)-β (R&D systems, Minneapolis, MN, USA), 20 ng/mL IL-6 (BioLegend, San Diego, CA, USA), 5 μg/mL anti-IFN-γ (BioLegend) and 5 μg/mL anti-IL-4 (BioLegend). Cells were cultured in this conditioned medium for 3 days. At day 0, tIK protein or phosphate-buffered saline (PBS) as a control was added.

### 4.3. Immunoblot Analysis

Cells were centrifuged at 1200 rpm at 4 °C for 10 min to collect cells. Cell pellets were lysed with radioimmunoprecipitation assay (RIPA) buffer (Santa Cruz, Dallas, TX, USA) containing a proteinase inhibitor cocktail (Thermo Fisher Scientific) and incubated for 10 min on ice. The lysed cell pellet was centrifuged at 12,500 rpm at 4 °C for 10 min to isolate the proteins. The concentration of isolated proteins was measured using the bicinchoninic acid protein (BCA) assay. Proteins were then separated by sodium dodecyl sulfate-polyacrylamide gel electrophoresis (SDS-PAGE) and transferred to polyvinylidenedifluoride membrane with a 0.2 μm pore size (Bio-Rad, Hercules, CA, USA). The target proteins were detected and analyzed by immunoblotting using the primary antibodies diluted in 0.1% PBS-Tween buffer containing 1% bovine serum albumin and 0.02% sodium azide. These primary antibodies included anti-A20 (1:1000) (Cell Signaling, Danvers, MA, USA) and anti-GAPDH (1:2000) (Enzo Life Science, Farmingdale, NY, USA).

### 4.4. Flow Cytometry

The splenocytes from the mice used in the in vivo experiments or CD4^+^ T cells differentiated to Th17 cells in vitro were stained to identify the cytokines within the cells. Prior to the intracellular staining, the cells were stimulated with phorbol 12-myristate 13-acetate (PMA) (Sigma-Aldrich, St Louis, MO, USA) (50 ng/mL) and ionomycin (Sigma-Aldrich) (200 ng/mL) for 3 h. For effective staining of cytokines, GolgiPlug (BD Biosciences, San Jose, CA, USA) was added to the cells with PMA and ionomycin as a protein transport inhibitor. After stimulation, cells were surface stained with antibodies to mouse CD4 (eBioscience) and then intracellular staining was performed with antibodies to mouse IFN-γ (BD Biosciences) and IL-17A (BD Biosciences) using the Foxp3/Transcription Factor Staining Buffer set (eBioscience). Before and after each staining step, cells were washed twice with wash buffer comprising 3% fetal bovine serum and 0.02% sodium azide in PBS. To prevent nonspecific binding, purified rat CD16/CD32 anti-mouse (Fc block, BD Biosciences) was added to the staining solution. The stained cells were analyzed using a FACS Canto II flow cytometer (BD Biosciences) and FlowJo software (Treestar, Ashland, OR, USA).

### 4.5. Enzyme-Linked Immunosorbent Assay (ELISA)

To detect IL-17 produced by Th17 cells, cells were stimulated with 1 μg/mL anti-CD3 (eBioscience) and 1 μg/mL anti-CD28 (eBioscience) for 24 h. To detect other cytokines produced by the CD4^+^ T cells, cells were cultured with 1 μg/mL anti-CD3 and 1 μg/mL anti-CD28 for 8 h or 24 h. Culture supernatants were collected at the relevant times. To measure the concentration of cytokines in culture supernatants we used ELISA kits for IFN-γ (eBioscience) and IL-17 (R&D Systems) according to the manufacturers’ instructions. We analyzed the cytokine concentrations using microplate reader software (Thermo Fisher Scientific).

### 4.6. RNA Isolation and Quantitative Polymerase Chain Reaction (qPCR)

Total RNA was isolated from mouse splenocytes using TRIzol reagent (Invitrogen) and used as the template for cDNA synthesis using a High-Capacity cDNA Reverse Transcription Kit (Applied Biosystems, Waltham, MA, USA). Quantitative PCR was performed using an iCycler MyiQ Single-Color Real-Time PCR Detection instrument (Bio-Rad) and SYBR Premix Ex Taq II (Takara, Shiga, Japan). We measured the expression level of mRNAs for IL-17A, IL-22, and IL-23 with 18s rRNA as the internal control. The following primers were used for qPCR: for IL-17A, 5′–ATC-CCT-CAA-AGC-TCA-GCG-TGT–3′ (forward) and 5′–GGG-TCT-TCA-TTG-CGG-TGG-AGA-G–3′ (reverse); for IL-22, 5′–TCA-TCG-GGG-AGA-AAC-TGT-TC–3′ (forward) and 5′–CAT-GTA-GGG-CTG-GAA-CCT-GT–3′ (reverse); for IL-23, 5′–TCC-TCC-AGC-CAG-AGG-ATC-ACC-C–3′ (forward) and 5′–AGA-GTT-GCT-CCG-TGG-GC–3′ (reverse); for 18s rRNA, 5′–CGG-ACA-GGA-TTG-ACA-GAT-TG–3′ (forward) and 5′–CAA-ATC-GCT-CCA-ACT-AA–3′ (reverse).

### 4.7. Animals

All animal studies were approved and performed according to the Institutional Animal Care and Use Committee of the Sungsim Campus at the Catholic University of Korea (IACUC Board Regulations #2016-009, Approved 27 May, 2016). Seven-week-old, male DBA/1J mice were purchased from Taconic and six mice per cage were maintained in a climate-controlled environment (temperature: 22 ± 1 °C, humidity: 55 ± 10%) and a 12 hour light/dark cycle.

### 4.8. Arthritis Model

We generated the CAIA mouse model of RA by intravenous injection of a cocktail of monoclonal antibodies against type II collagen into DBA/1J mice. At day 3 after the monoclonal antibody injection, we injected LPS 0114:B4 intraperitoneally to stimulate the induction of RA. We purchased the monoclonal antibodies against type II collagen and LPS 0111:B4 from Chondrex (Redmond, WA, USA) and followed the manufacturer’s instructions. We checked the paws of the mice daily for the severity of arthritis. The severity was scored on a scale of 0 (no symptoms) to 4 (maximum level of symptoms) and the scores of all four limbs were summed to calculate the score for each mouse. The severity was scored under the state of blinding the experimental groups using the following guidelines: score 0, no symptoms such as swelling or erythema; score 1, mild erythema and swelling on toes; score 2, noticeable erythema and swelling on toes and soles; score 3, moderate erythema and swelling on entire paw from ankle to toes; and score 4, maximal erythema and swelling on entire paw from ankle to toes.

Mice experiments were carried out in accordance with the relevant guidelines and regulations established by the ethical guidelines and regulations of the Korean Association for Laboratory Animals [48]. All experimental techniques/procedures were approved by the Institutional Animal Care and Use Committee of the Songsim Campus at the Catholic University of Korea (IACUC Board Regulations #2016-009, Approved 26 May, 2016).

### 4.9. Treatment of Animals

Mice were injected daily intraperitoneally (i.p.) with tIK protein (10 mg/kg) in PBS as a vehicle. As a positive control, etanercept (commercial name; Enbrel, 10 mg/kg) was injected i.p. every 3 days.

### 4.10. Histopathology of Arthritis

For histological analysis, the joint tissues of mice were fixed in neutral 10% formalin solution (Sigma) and decalcified using a routine buffer (14% free acid EDTA, pH 7.2) before the tissue was embedded in paraffin blocks and sectioned onto slides, stained with hematoxylin and eosin (H&E) and for immunohistochemistry using Vectastain ABC kit (Vector Laboratories, Burlingame, CA, USA). The stained slides were viewed and analyzed under a light microscope (Axio imager D2, Zeiss, Oberkochen, Germany).

### 4.11. Plate Array for Profiling of Transcription Factor Activation

Nuclear extracts were prepared from splenocytes treated with tIK protein, using a commercial kit (Signosis, Santa Clara, CA, USA). The nuclear extracts were used as samples for a plate array to monitor the activation of transcription factors (Signosis). The results of the array were detected by a luminometer (Promega GloMax Explorer, Promega, Madison, WI, USA) and analyzed according to the manufacturer’s guidelines.

### 4.12. Statistical Analysis

Statistical analysis of the data was conducted using Prism 5 software (GraphPad Software, San Diego, CA, USA). All values are expressed as the mean ± standard deviation (SD). The significance of differences between groups was analyzed by the students’ *t*-test. Differences where *p* < 0.05 were considered significant.

## Supplementary Materials

Supplementary materials can be found at www.mdpi.com/1422-0067/18/9/1976/s1.

## Abbreviations

AP	activator protein
BCA	bicinchoninic acid protein
CAIA	collagen antibody-induced arthritis
cAMP	cyclic AMP
CBF	core binding factor
CIA	collagen-induced arthritis
CIITA	class II transactivator
CVB3	Coxsackievirus B3
HIF	hypoxia inducing factor
H&E	hematoxylin and eosin
IFN-γ	interferon-γ
IL	interleukin
IL1RaKO	interleukin-1 receptor antagonist knockout
i.p.	intraperitoneally
IRF	interferon regulatory factor
LPS	lipopolysaccharide
MACS	magnetic activated cell sorting
MHC	major histocompatibility complex
NFAT	nuclear factor of activated T cells
PBS	phosphate-buffered saline
PMA	phorbol 12-myristate 13-acetate
qPCR	quantitative polymerase chain reaction
RA	rheumatoid arthritis
RIPA	radioimmunoprecipitation assay
SDS-PAGE	sodium dodecyl sulfate-polyacrylamide gel electrophoresis
Tg	transgenic
TNF	tumor necrosis factor
WT	wild-type
