# Supplementary material for: Therapeutic Effect of Exogenous Truncated IK Protein in Inflammatory Arthritis
Source: Int J Mol Sci. 2017 Sep 14;18(9):1976. doi: 10.3390/ijms18091976 (PMC5618625; doi:10.3390/ijms18091976)
Supplement: Supplementary file 1 [file ijms-18-01976-s001.pdf]

Supplementary Materials: Therapeutic Effect of Exogenous Truncated IK Protein in Inflammatory Arthritis

Seulgi Choi [1,†], HyeLim Park [1,†], SeoYeon Jung [1], Eun-Kyung Kim [2], Mi-Ra Cho [2], Jun-Ki Min [3], Su-Jin Moon [3], Sang-Myeong Lee [4], Jang-Hee Cho [5], Dong-Hee Lee [5], Jae-Hwan Nam [1,*]

Nucleotides sequence

CTCATGACAGCTGATAACAAGTTCAACAAAGAGCAGCAGAACGCATTCTACGAGATCTTGCATCTGCCGAATTTAAACGAAGAA
CAGCGTAACGGTTTCATCCAGTCTCTGAAGGATGACCCGAGCCAGAGCGCTAACCTGCTGGCTGAGGCTAAGAAACTGAACGA
CGCTCAGGCGCCAAAGGCCATGATGAACATTTTTGAAGACATTGGGGATTACGTTCCTTCTACAACCAAGACACCTCGGGACAA
GGAACGTGAGAGATACCGGGAACGTGAACGTGATCGGGAACGGGACAGAGACAGGGAGCGAGACAGGGAGCGAGACCGTG
AGAGGGAGAGAGAGCGAGACCGGGAACGGGAACGAGAGGAGGAAAAGAAAAGGCACAGCTACTTTGAGAAGCCAAAAGT
GGATGATGAGCCCATGGATGTTGACAAAGGACCTGGATCTGCAAAAGAGTTGATCAAGTCCATCAATGAAAAATTCGCTGGGTC
TGCTGGCTGGGAAGGCACTGAATCGTTGAAGAAGCCAGAAGATAAGAAGCAGCTGGGCGATTTCTTTGGCATGTCCAACAGTT
ACGCAGAATGCTATCCAGCCACGATGGATGACATGGCTGTAGATAGTGATGAAGAGGTAGATTATAGCAAAATGGACCAGGGTA
ACAAGAAGGGTCCCTTAGGCCGCTGGGACTTCGATACTCAGGAGGAATACAGCGAGTACATGAACAACAAGGAGGCTCTGCCC
AAGGCTGCATTCCAGTATGGCATCAAGATGTCTGAAGGACGGAAAACCAGACGATTCAAAGAAACCAATGATAAGGCAGAGCT
TGATCGACAGTGGAAGAAAATAAGTGCAATCATTGAGAAGAGGAAGAGGATGGAAGCAGATGGGGTCGAAGTGAAAAGACC
AAAGTACTAA

Amino acids sequence

LMTADNKFNKEQQNAFYEILHLPNLNEEQRNGFIQSLKDDPSQSANLLAEAKKLNDAQAPKAMMNIFEDIGDYVPSTTKTPRDKE
RERYRERERDRERDRDRERDRERDRERERERDRERERERDRERERERDRERERERERDRERERERERDRERERERERERERERD
ESLKKPEDKKQLGDFFGMSNSYAECYPATMDDMAVDSDEEVDYSKMDQGNKKGPLGRWDFDTQEEYSEYMNNKEALPKAAFQ
YGIKMSEGRKTRRFKETNDKAELDRQWKKISAIIEKRKRMEADGVEVKR PKY

IgG binding domain of protein A   Truncated IK (tIK)

Figure S1. Nucleotide and amino acid sequences of tIK fused with IgG binding domain.

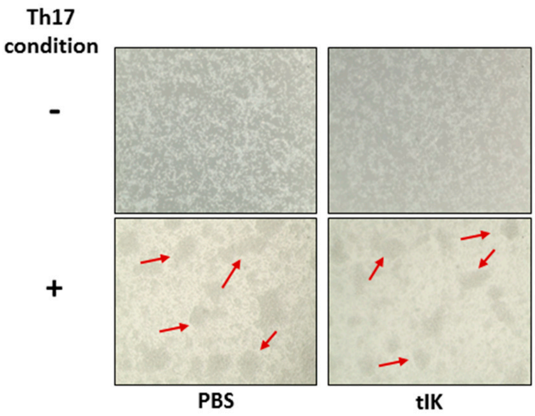

Figure S2. Cell clumps seen in Th17-polarizing medium. $CD4^+$ T cells isolated from 7-week-old Balb/c mice using magnetic activated cell sorting were cultured in Th17-polarizing medium or control medium. After 3 days, the cells formed clumps only in Th17-polarizing medium. The red arrows point to the clumps.

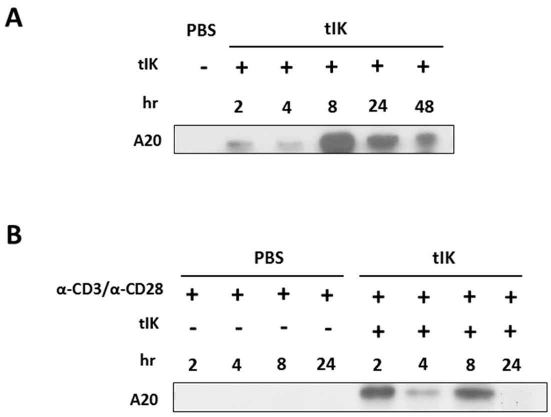

Figure S3. The time course of A20 induction by treatment with exogenous tIK protein. (A) CD4+ T cells isolated and cultured as described for Figure 4(A). At 2, 4, 8, 24, and 48 h posttreatment with tIK protein, the cells were harvested and lysed using RIPA buffer containing a protease inhibitor cocktail. A20 was detected in the total extracted protein by Western blot analysis. (B) CD4+ T cells were isolated and stimulated as described for Figure 4(B). At 2, 4, 8, and 24, h posttreatment with tIK protein, total protein from cells were extracted and used for Western blot analysis as described for Supplementary Figure 3(A).

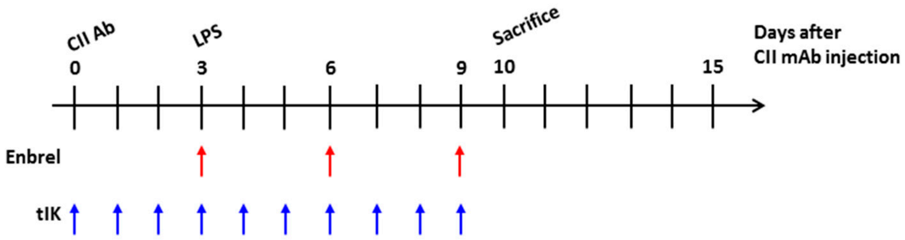

Figure S4. Detailed schedule for induction of CAIA in mice, and the schedule for treatments with tIK protein or Enbrel. CAIA was induced by an intravenous injection of monoclonal antibody against type II collagen into DBA/1J mice. At day 3 after the monoclonal antibody injection, LPS was injected to stimulate the. PBS (vehicle) or tIK protein (tIK) was injected every day; Enbrel was injected every 3 days.

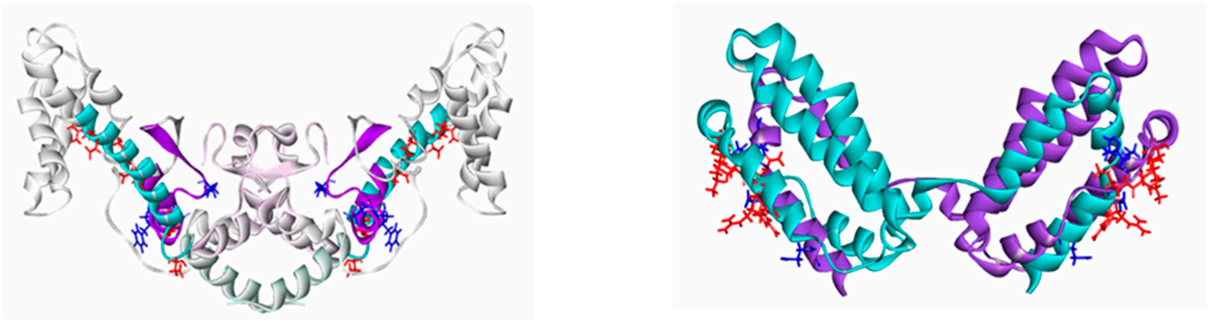

tIK protein IL-10

Figure S5. 3D structure analysis of tIK protein**.** The 3D structure of tIK protein and IL-10 dimer were analyzed *in silico* using PyMol Molecular Graphic System (DeLano Scientific LLC).
